# Electromagnetic wave absorption and structural properties of wide-band absorber made of graphene-printed glass-fibre composite

**DOI:** 10.1038/s41598-018-30498-3

**Published:** 2018-08-13

**Authors:** F. Marra, J. Lecini, A. Tamburrano, L. Pisu, M. S. Sarto

**Affiliations:** 1 Department of Astronautics, Electrical and Energy Engineering, Sapienza University of Rome, 00184 Rome, Italy; 2 Research Center for Nanotechnology applied to Engineering, Sapienza University of Rome, 00184 Rome, Italy; 3 Leonardo S.p.A, Aircraft Division, Corso Francia 426, Torino, Italy

## Abstract

Lightweight composites combining electromagnetic wave absorption and excellent mechanical properties are required in spacecraft and aircraft. A one- dimensional metamaterial absorber consisting of a stack of glass fibre/epoxy layers and graphene nanoplatelets/epoxy films was proposed and fabricated through a facile air-spraying based printing technology and a liquid resin infusion method. The production process allows an optimum dispersion of graphene nanoplatelets, promoting adhesion and mechanical integration of the glass fibre/epoxy layers with the graphene nanoplatelets/epoxy films. According to experimental results, the proposed wide-band absorber provides a reflection coefficient lower than −10 dB in the range 8.5–16.7 GHz and an improvement of flexural modulus of more than 15%, with a total thickness of ∼1 mm. Outstanding electromagnetic wave absorption and mechanical performance make the proposed absorber more competitive in aeronautical and aerospace applications.

## Introduction

In the era of digitalization, wireless devices and industry 4.0, the control of electromagnetic (EM) disturbances and the reduction of radiated field pollution is crucial in order to prevent EM interference and safety issues. A lot of research has been carried out on the development of high-performing multifunctional absorbers. Moreover, the use of EM wave absorbers covers a wide range of applications in defence systems such as reduction of radar cross-section (RCS). In aircraft or spacecraft, reduction of weight is a priority, which can be achieved through the development and use of lightweight multifunctional materials, combining EM shielding and wave absorption properties with structural strength and mechanical resistance.

The attenuation of EM waves can be achieved by reflection or absorption of the received radiation^1^. Generally, EM waves are shielded by means of conventional metal screens, which possess drawbacks of increased weight, reduced flexibility, susceptibility to corrosion, and restricted effectiveness of tuning the EM radiation. Jaumann absorbers^2^ and Salisbury screens^3^ are very good examples of radar absorbers. Multilayer Jaumann absorbers suffer from large thickness and bulkiness as each layer produces a single narrow band resonance. Recently, metamaterials (MMs) have attracted much attention owing to their ability to manipulate EM waves^4–8^. Moreover, polymer composites filled with conducting micro/nanoparticles, have gained attention due to their light weight, flexible design and corrosion-resistant properties^9–11^.

In recent years, carbon-based nanofillers have been widely used in material science due to their modification properties of the matrix in multifunctional composites^11^. During the last decade, several studies^12–17^ have demonstrated the potentiality of graphene nanoplatelets (GNP) as nanofiller in thermosetting composites with tailored EM properties for application in radar absorbing materials. Lv *et al*. presented a cement-based composite filled with GNP and hollow glass microspheres, with improved mechanical and EM properties^18^. The produced composite showed an average reflectivity loss of −8.2 dB in the range of 2–18 GHz. Chandrasekaran *et al*. reported a production process of GNP/epoxy nanocomposites with a filler content up to 2%wt^19^ demonstrating enhanced electrical, thermal and mechanical properties.

Specific interest has been also addressed towards the development of structural polymer composites with improved properties through the addition of carbon nanomaterials. Within this context, several studies have been focused on glass-fibre or carbon-based fibre reinforced composites, which are widely used in the aeronautical industry for structural components. An extensive review on the use of carbon-based nano-reinforcement to improve the degradation properties of laminated continuous-fibre/epoxy composites is reported by Lubineau and Rahaman^20^. Umer *et al*. presented a production method of graphene oxide (GO)-reinforced glass-fibre composite material with enhanced flexural modulus and flexural strength, up to the 21% and the 30%, respectively, for a GO content of 0.25%^21^. GO was dispersed in the epoxy resin and the resulting polymer mixture was used to produce the glass-fibre composite through liquid resin infusion. A critical issue is the control of nanofiller agglomerates along the fibre texture. The reinforcement effect of GO in glass fibre/epoxy composites was also investigated by Prusty *et al*.^22^, who dispersed GO in the epoxy resin, and the resulting polymer mixture was used to impregnate the glass-fibre texture and produce the composite through hand lay-up process. The use of GNP as reinforcement in glass fibre/epoxy composites was investigated by Kamar *et al*.^23^. A suspension of GNP in isopropanol was brushed over the glass fabric. The resulting coated fabric was then used to produce the laminate composite through vacuum-assisted resin transfer moulding, after solvent evaporation. Zhang *et al.* proposed a spray-coated carbon nanotubes (CNT) on carbon-fibre prepreg to improve the fracture toughness of the composite^24^. The use of GO as filler in carbon fibre composites was also exploited in order to improve the interfacial and mechanical properties of the material^25,26^. Moreover, a graphene/epoxy interleave was proposed for delamination toughening of carbon fibre/epoxy composites^27^. However, in all these studies there is not a focus on the improvement of the EM properties of the composite for the development of radar absorbing structures (RAS).

Nevertheless, there are some studies that investigate the combined enhancement of both the mechanical and electrical properties of the composite through the addition of carbon-nanofiller. Qin *et al*. reported an improvement of the mechanical and electrical characteristics of carbon-fibre composite through the use of GNP-coated carbon fibre in prepreg-laminated composites^28^. However, the developed material is not suitable for application in RAS since it is too conductive and it cannot provide EM absorption.

As regards the development of carbon-based polymer composites for application as radar absorbing material, a crucial aspect is to obtain materials with a loss tangent (defined as the ratio between the modulus of the imaginary and real part of the effective dielectric permittivity) greater than 0.5. This can be achieved in epoxy based composite through the combined use of micro- and nanofillers^13^, as well as through the use of 2D-carbon nanostructures, like GNP, having thickness in the nanometre range and lateral dimensions up to tens of microns^12,14,15^. Nevertheless, the production of structural fibre-reinforced composite laminates with radar absorbing properties is still an open issue because the amount of carbon nanofiller, necessary to obtain the desired value of electrical conductivity, induces a deterioration of mechanical properties of the composite. In fact, nanostructures tend to agglomerate and to form aggregates along the fibre fabric. This implies that the resulting composite is poorly conducting, characterized by a relatively low loss tangent and full of defects made of nanofiller-based macro-agglomerates. Actually, it was demonstrated by Acquarelli *et al*.^29^ that GNP agglomerates in thermosetting composites behave as mechanical defects and induce the formation of cracks, with consequent degradation of the mechanical properties of the composite.

Therefore, the design and processing of high-performance EM-absorbing materials based on CNT and GNP, with improved mechanical properties and wide-band absorption remain a tough challenge.

In this paper, we propose an innovative approach in order to produce a high performance wide-band RAS based on GNP. The absorber has the structure of a 1D-metamaterial made of a stack of 4 periods of glass-fibre/epoxy composite and of GNP/epoxy films, and it is produced through vacuum assisted resin infusion (VARI). The periodic multi-layered structure of the absorber enables to achieve high EM-wave absorption and at the same time high mechanical strength, with a total GNP content which is limited to 1 or 2%wt. GNP that are sprayed over the glass fibre fabric through a facile air-spraying method, play the role of sizing agent and lossy filler. A full characterization of the morphological, mechanical, electrical, and electromagnetic properties of the produced glass-fibre composites filled with GNP was performed. The novelty of this work is twofold. First, it provides the design of a new RAS, which consists of a sub-resonant 1D-metamaterial enabling the wide-band response with reduced thickness. Second, it describes the fabrication process we developed to obtain a composite characterized by both high mechanical strength and radar absorbing properties. These two aspects enable to overcome the limitation of state-of-art RAS for aeronautical applications.

## Results

GNP, dispersed in a mixture of acetone and epoxy resin were printed by air-spraying over the glass-fibre (GF) fabric. The spray-coating step was performed over a single face or on both faces of the GF fabric, thus obtaining S-type or D-type composites. After the pre-wetting of the GF-fabric with the GNP-filled sizing agent, the plies were stacked in order to form a laminate, having a thickness of 1.0–1.1 mm, depending on the amount of GNP sprayed over the fabric surface. At this point, the composite RAS is produced applying the infusion system^30–32^. Illustration of the fabrication process for production of the composite can be found in the section “RAS Production Process”. Specimens with increasing GNP content up to ∼3%wt were produced, as listed in Table 1.

**Table 1:** Produced composite samples: concentration of GNP in the glass/epoxy composite and measured flexural modulus and DC electrical conductivity.

Sample	GNP		Sprayed face(s)	Flexural Modulus (GPa)	DC electrical conductivity (S/m)
	%wt.	g/m^2^			
Neat	—	—	—	18.42	—
S1	0.55	2.5	Single	16.96	0.24
S2	0.99	4.5	Single	17	0.41
S3	1.54	7.0	Single	16.54	3.41
S4	1.97	9.0	Single	16.4	5.27
S5	2.96	13.5	Single	17.11	11.61
D1	1.10	2.5 (×2)	Double	21.38	0.18
D2	2.19	5.0 (×2)	Double	19.13	4.60

**Figure 1:**
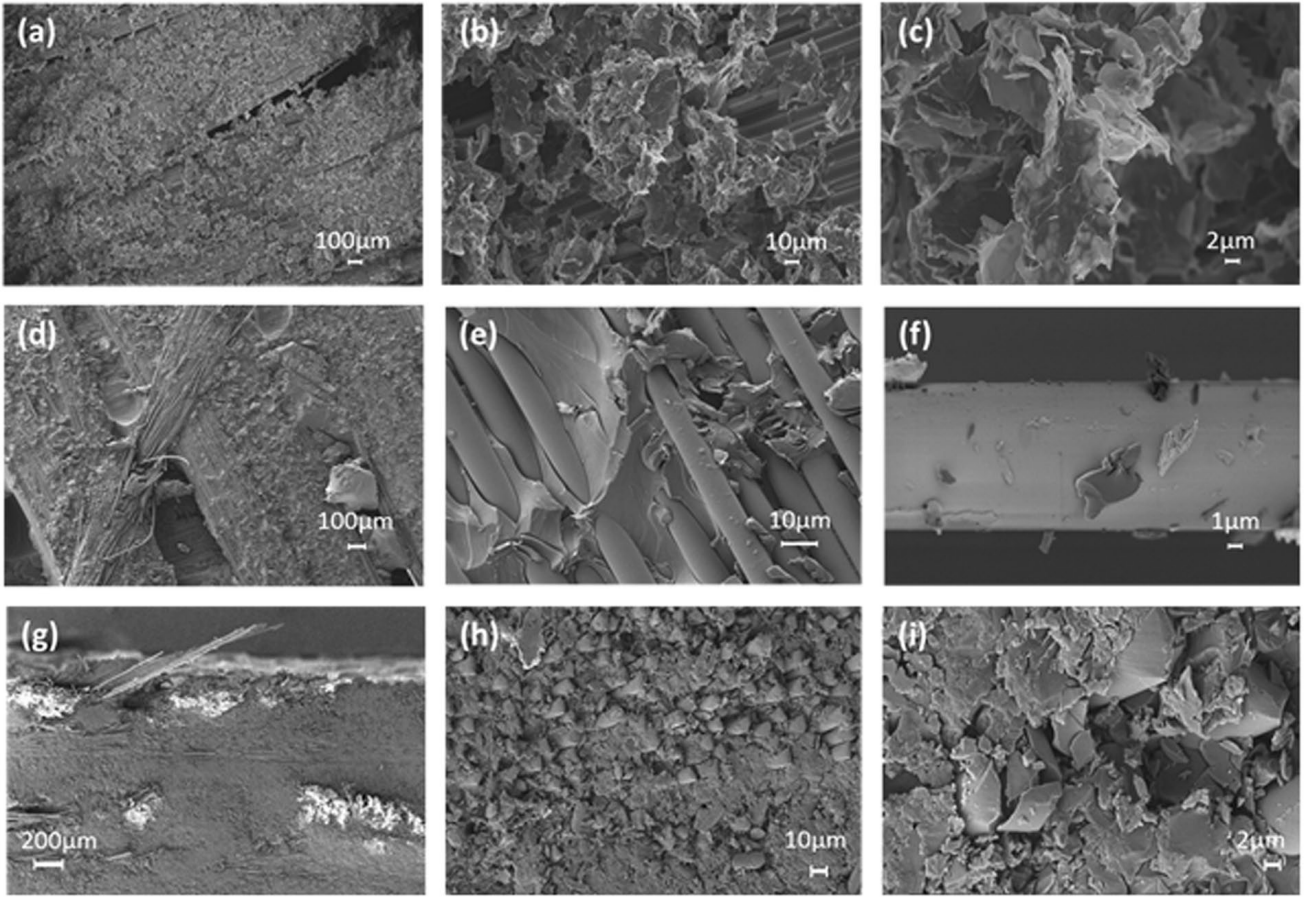
SEM images: (**a**–**c**) the top face of a glass fibre ply with GNP-based sizing agent, before VARI; (**d**–**f**) the top face of sample S1 at different magnification; (**g**–**i**) the fracture-section of sample S1 at different magnification.

Figure 1(a–c) shows the details of the surface of the glass fibre ply, after spraying with GNP-based sizing agent and before the infusion process. The SEM images demonstrate the homogeneous and total covering of the tissues, at different magnifications 100X, 500X, 1.0KX, 5.0KX.

It is observed that the glass fibre texture is covered by a thin layer of GNP-epoxy composite and that GNP infiltrate among fibres due to their small dimensions. This is also confirmed by SEM analysis of the cured composite. Figure 1(d–f) shows the surface of the produced composite at different magnifications. We observe that GNP are deposited over the surface of the fibres (Fig. 1d), but also that they are infiltrated among fibres, being well integrated in the epoxy matrix (Fig. 1e). In the largest magnification (Fig. 1f), there appears the detail of GNP which are distributed all around the fibre, thus confirming the hypothesis that the nanofiller is well dispersed and integrated inside the composite.

Next, the cross-section of the RAS was analysed, in order to better investigate the integration of filler, resin and glass fibres. To this purpose, the samples were broken in liquid nitrogen. Figure 1(g–i) show the SEM images of the cross-section of the panel with exposure of the glass fibres. We notice the thin GNP-epoxy coating over the top face of the laminate (Fig. 1g) and the penetration of GNP in the inner volume of the composite (Fig. 1h,i).

In conclusion, from the analysis of the SEM images, it results that GNP sprayed over the GF texture surface penetrate into the texture after liquid resin infusion of the epoxy resin and complete curing. The penetration depth can reach 100–150 µm, depending on spraying condition, liquid resin infusion process and GNP concentration. The details at higher magnifications also demonstrate a good interaction between fibres, resin and GNP. Therefore, we conclude that during the VARI process GNP remain attached to the fibres and do not agglomerate in large aggregates.

The results of the dc electrical conductivity measurements are reported in Table 1. We notice an increase in the dc electrical conductivity as function of the GNP concentration in all laminate composites. Moreover, it results that S-type and D-type samples having nearly the same concentration of GNP are characterized by similar values of dc conductivity. This demonstrates that GNP are uniformly distributed in both composite types.

**Figure 2:**
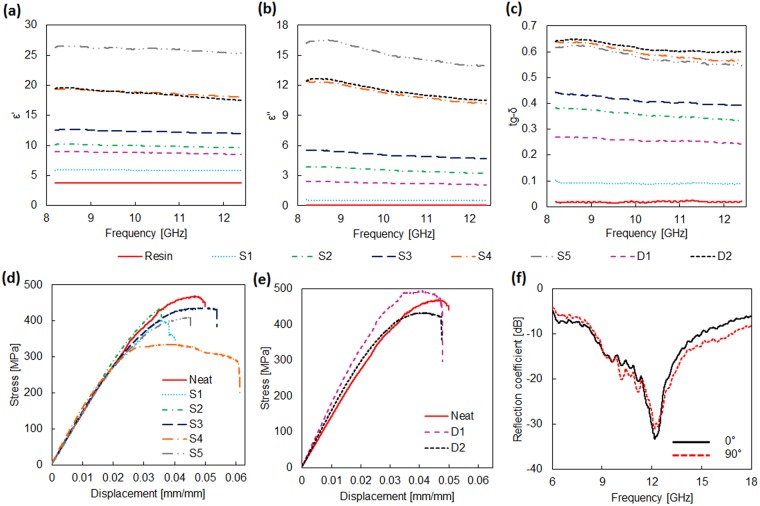
(**a**) Real part *ε*′, (**b**) imaginary part ε″ of the complex effective permittivity and (**c**) loss tangent (tan δ) of the effective complex permittivity of the produced composite samples. (**d**), (**e**): Stress-strain curve of the produced S-type and D-type composites. (**f**) Measured frequency spectrum of the reflection coefficient for 0°- and 90°-polarizations of the RAS obtained by layering 4 plies of composite D1 (having thickness of 1 mm) over an aluminium plate (2 mm in thickness).

The electromagnetic characterizations were performed after conditioning the samples in a temperature/ humidity controlled environment for 24 h. The measured real part (*ε*′) and imaginary part (*ε*″) of the complex effective permittivity of the GNP-filled glass fibre/epoxy composite are reported in Fig. 2(a,b), respectively, from 8.2 GHz up to 12.4 GHz. The results are obtained averaging the data from six different measurements. A slight increase of data dispersion is observed especially at higher filler content. In particular, the maximum value of the standard deviation of *ε*′ and *ε*″ over the considered frequency range is respectively ±0.03 and ±0.06 for S1-type samples, ±1.41 and ±1.11 for the ones with highest GNP concentration (S5). It can also be noted that both *ε*′ and |*ε*″| increase with the filler weight fraction whereas decrease with the frequency. Moreover, the samples S4 and D2, which have nearly the same filler concentration, are characterized by very similar values of the complex effective permittivity, as also obtained by the dc conductivity tests.

Finally the loss tangent (tan *δ*) of the produced composite samples, which is representative of the power loss in the material with respect to the stored reactive power, is estimated as the ratio between imaginary and real part of the complex effective permittivity. The computed values of tan *δ* are reported in Fig. 2(c). We notice that tan *δ*, which increases with the concentration of GNP in the composite, is always greater than 0.58 in the whole frequency range for samples S4, S5, D2.

The mechanical characterizations of the produced samples were performed in order to study the flexural stress-strain curve, shown in Fig. 2(d,e). It can be noted that the D-type samples (Fig. 2e) perform better than the S-type ones (Fig. 2d), and that they are characterized by higher values of the flexural modulus than the neat composite (Table 1). This is due to the fact that in the D-type samples the sizing agent containing GNP is sprayed over both faces of the glass fibre texture so that GNP are better integrated within the composite and slippage of the fibre plies is inhibited. From the data of flexural modulus reported in Table 1 we also notice that S-type samples have modulus values slightly lower than the one of the neat resin sample. The corresponding data shown in the Table reveal that the flexural modulus of S-type composites is reduced by 7–11% due to the presence of GNP, whereas it increases by 16% for sample D1 (having nearly the same amount of GNP of sample S2) and by 3.9% for sample D2 (having nearly the same amount of GNP of sample S4).

Finally, the measured reflection coefficient of the RAS obtained by layering 4 plies of the composite D1 over an aluminum plate of thickness 2 mm is shown in Fig. 2(f). The total thickness of the composite is ∼1 mm and the minimum reflection coefficient reaches the value of −34.5 dB at the frequency of 12.16 GHz. The bandwidth at −10 dB is of ∼7.2 GHz, which corresponds to the 60% of the central frequency. Measurements performed in both polarizations show a good isotropic response of the panel, which makes it suitable for aviation purposes.

## Discussion

**Figure 3:**
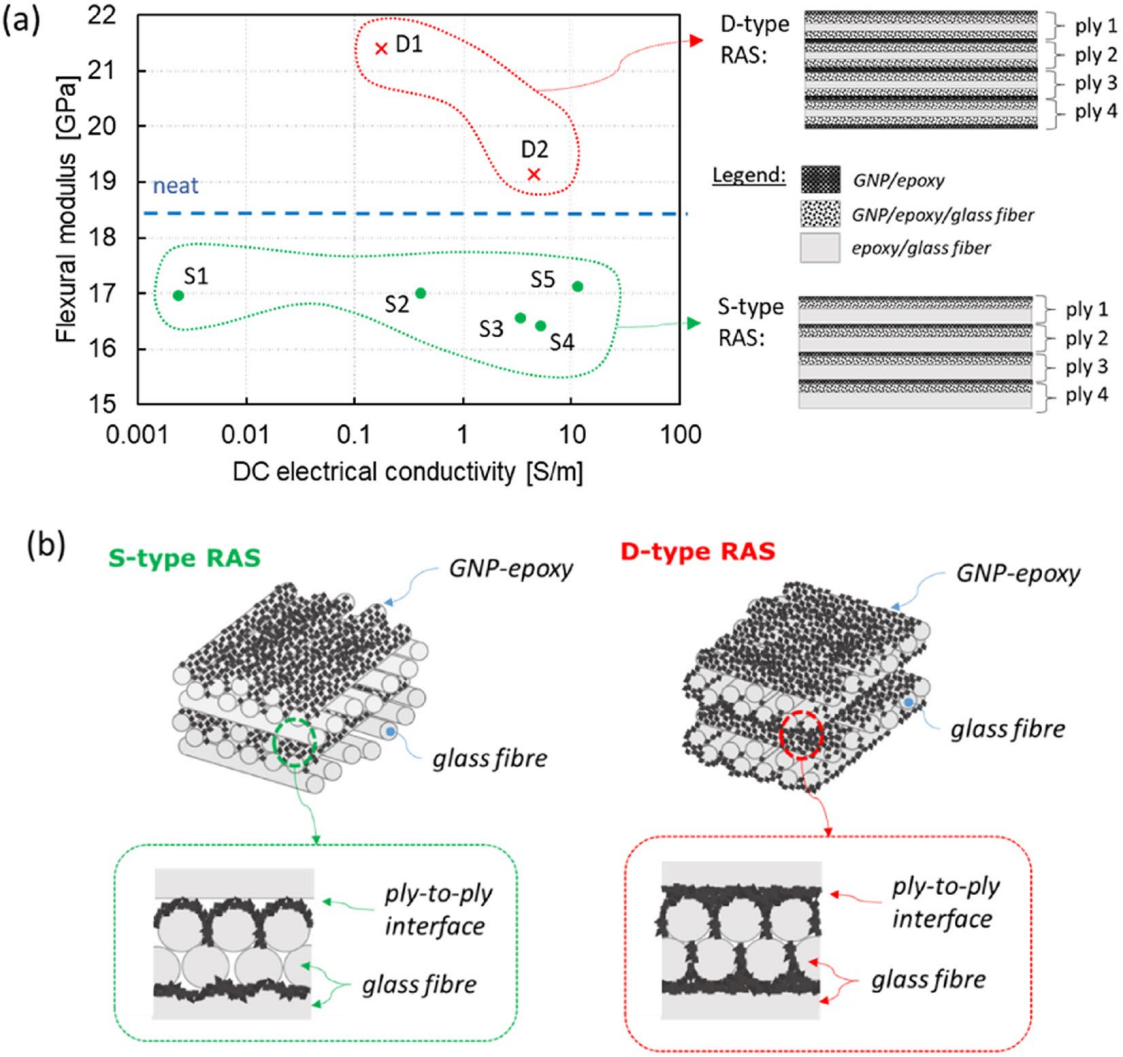
Ply-to-ply interface in S-type and D-type composites; Flexural modulus versus DC electrical conductivity of the produced GNP filled glass fibre/epoxy composites.

This paper proposes an innovative approach in order to produce a highly performing wide-band RAS based on GNP, having the structure of a 1D-metamaterial made of a stack of 4 periods of GF/epoxy composite and of GNP/epoxy films. The GNP-filled GF/epoxy composite was designed as structural material, having potential application in the aerospace, automobile, marine, and wind energy industries. The ability to directly spray-coat the graphene fillers on to the GF fabric could make this concept particularly suitable for large-scale industrial applications and it is compatible with existing structural composite processing technologies. The proposed method not only overcomes viscosity issues in traditional resin loaded approaches^21,22^, when the nanofiller is dispersed in the epoxy resin used to impregnate the fibre texture, but it also allows to obtain a fine spatial control of GNP distribution and dispersion in the composite. Moreover, in the proposed method, the use of GNP dispersed in a mixture of acetone and epoxy resin as sizing agent enables to produce a composite with enhanced mechanical and electrical properties. As shown in the results, a weight fraction of GNP of 1%wt leads to an increase of 16% of the flexural modulus for the D-type specimen or to a minimum reduction of only 7% of the flexural modulus for the S-type specimen. This represents a clear novelty with respect to a similar approach which, using the same GNP concentration of 1%wt caused a reduction of more than 50% of the material flexural modulus^23^. The 1D metamaterial structure consisting in a 4 period-stack of GF/epoxy composite and GNP/epoxy film enables to obtain wide-band EM wave absorption in the rage 8–18 GHz, even for a very low content of GNP, and at the same time an improvement of the mechanical properties, as summarized in Fig. 3(a). The improvement of the interfacial ply-to-ply interaction in the D-type composite is illustrated in Fig. 3(b). The GNP-epoxy sizing agent sprayed over the fibre fabric improves the adhesion between fibres of facing plies and inhibits slippage of the fibre levels.

Finally, the method proposed in this paper is applied to the fabrication of a thin wideband RAS, having in the X band (8.2–12.4 GHz) and Ku band (12.4–18 GHz) a minimum reflection of −34.5 dB and a bandwidth at −10 dB equal to the 60% of the central frequency. To the best of our knowledge, this is the first example of a wideband thin RAS made of GNP-filled GF/epoxy composite laminate, produced through liquid resin infusion.

## Methods

### Materials

The resin used, was single part epoxy, with an optimized low viscosity/temperature profile allowing injections at temperature of 70 °C. The initial injection viscosity is around 0.1 Pa·s, the curing time is two hours at the temperature of 180 °C. Acetone (C3H6O) was 99.9% of purity (Sigma-Aldrich CoLtd). The glass fibre fabric was a commercial product manufactured by SAERTEX; it has a weight of 301 g/m^2^ and consists of two layers of E-glass fibre laid at ±45° to each other; the two superimposed layers are sewn together with a polyester thread. The dry fibres fabric has a thickness of ~250 µm. GNP used in this study are 2D-carbon nanostructures consisting of small stacks of graphene obtained by exfoliation of expanded graphite. They are characterized by a thickness of ∼20–30 nm and a lateral dimensions of up to a few of microns.

### GNP-printing over the GF fabric

**Figure 4:**
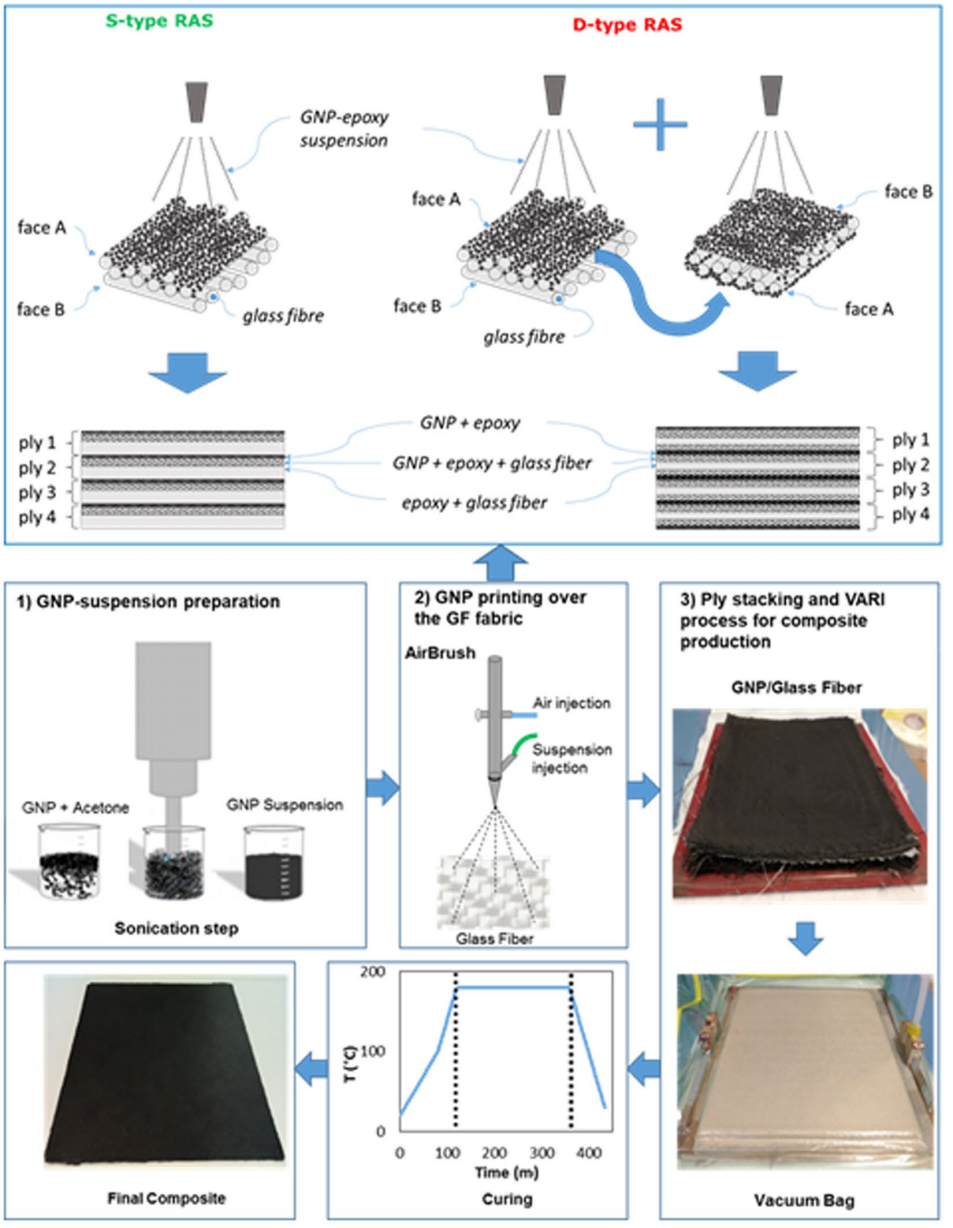
Schematic of the production process.

The commercial glass fibre fabric was cut in square patches with dimensions 31 × 31 cm. GNP were firstly dispersed into acetone and sonicated using an ultrasonic probe. Next, the suspension was mixed with a solution of epoxy at 2% wt in acetone, acting as a sizing agent. Different weight concentration of GNP in the mixture were used. Successively, the GNP suspension in epoxy and acetone was air-sprayed over the GF-fabric. The spray-setup consists of a commercial airbrush, mounted over an XY-plotter, controlled through a PC. Two different sets of samples were produced, as sketched in Fig. 4. The S-type composite (left side of Fig. 4) was obtained by staking GF-fabrics sprayed with the GNP-epoxy suspension only on a single face. The D-type composite was obtained with GF-fabrics coated with GNP on both faces (right side of Fig. 4). The GNP content in the final composite is estimated according to the standard ASTM D-3171 (data in Table 1, column 2). Nevertheless, the different composite specimens produced are characterized in term of the amount (weight) of GNP deposited per-unit-square on a single face of each ply as reported in Table 1 (data in column 3).

### RAS Production Process

The composite laminates were produced through the VARI process. In order to ensure the Werren & Norris rule of isotropy, the following stacking angle of the plies was applied^32^:

$$\alpha =\frac{2\pi }{n}$$

in which α is the angle of misalignment of one fabric over the other and n is the number of stacked plies (n ≥ 3). A 4-ply layup was used to manufacture the test specimens.

After the pre-wetting of the GF-fabric with the GNP-filled sizing agent, the plies were stacked in order to form a laminate, having a thickness of ∼1.1 mm, depending on the amount of GNP sprayed over the fabric surface. At this point, it was possible to produce the composite applying the infusion system^31,33^. Therefore, the 4-ply stack was inserted in a vacuum bag. The edges of the flexible sheet were sealed against the mould to form a sealed envelope surrounding the lay-up.

The epoxy resin, pre-conditioned at 70 °C in order to avoid formation of air-bubbles, was introduced into the envelope at 100 °C in order to increase the wettability. In the meanwhile, the vacuum was applied to the bag using a membrane vacuum pump at a pressure of about 0.9 bar. Curing was performed in oven at 180 °C for 2 hours. Then the mould was cooled at room temperature. The full production process is sketched in Fig. 4.

### Sample characterization

#### Microscopies

The morphology of the GF-fabric was investigated through electronic microscopy in order to assess the uniformity of distribution of the GNP-based sizing agent. The SEM images were performed using a Zeiss Auriga field-emission scanning electron microscopy (FE-SEM), available at Sapienza Nanotechnology and Nanoscience Laboratory (SNN-Lab). Samples to be imaged were bonded to SEM stubs using carbon double sided adhesive tape. Before the characterization, the samples were sputtered with a conductive layer of chrome of thickness ∼15 nm, in order to avoid electrostatic charging. The images were acquired using a 5 keV electronic beam acceleration voltage and a variable working distance between 7 mm and 8 mm.

#### Mechanical Characterization

The mechanical characterization of the produced samples was performed using a universal testing machine (Instron 3366) equipped with a 500 N load cell. The 3-point bending tests were carried out at a constant cross-head speed of 1 mm/min on unloaded GF composite samples and on GNP-filled specimens, according to the standard ASTM D790. The dimension of the beam-shaped specimens were 80 × 4 × ∼1.1 mm. The scope of the bending tests was to assess the influence of nanofiller concentration on the mechanical properties of the composites.

#### Electrical Characterization

The effective dc electrical conductivity (σ) of the produced samples was obtained from resistance (R) measured by the two-wire volt-amperometric method, applying the following expression:

$$\sigma =\frac{l}{wtR}$$

in which w, t and l are the width, thickness and length of the brick-shaped sample, which was contacted using a silver-based paint (Electrolube®) bonded to two metal wires through a conducting epoxy glue (Circuitworks®). All samples were dried for 24 h in a desiccator before the electrical tests. R was measured in a controlled environment (temperature of 23 ± 0.5 °C and humidity of 35 ± 5%), using a dc/ac current source (Keithley 6221) and a nano-voltmeter (Keithley 2182 A).

#### Electromagnetic Characterization

The relative effective complex permittivity of the different produced composites was extracted with the iterative modified Nicolson-Ross-Weir algorithm^13^ from the scattering parameters of specimens measured in the frequency range 8.2–12.4 GHz (X-band) using a vector network analyzer (Anritsu Vector Star MS4647A), rectangular waveguides (WR-90) and following the ASTM 5568 standard specifications. In particular, three brick-shape specimens, having dimensions 22.86 × 10.16 mm, were prepared for each composite type using a CNC milling machine (Proxxon FF 500 CNC) and then were inserted in aluminum flanges with WR-90 size. A total of six different measurements were performed on each batch and permittivity data were averaged, in order to reduce uncertainty.

#### Reflection coefficient measurement

The produced composite panel has a dimension of 30 × 30 cm after curing and edge trimming. The panel was then glued over a thick aluminum plate having the same dimensions, using a thin layer of epoxy-based glue. In order to measure the reflection coefficient, the resulting RAS was tested in the frequency range from 6 GHz to 18 GHz, satisfying far field conditions. The set-up was based on a pair of properly designed wide-band horn antennas, having circular aperture of 5.5 cm, connected to the two ports of the Anritsu Vector Star MS4647A^13,15^. The reflection measurements were performed for normal incidence in bi-static mode.
